# Convolutional neural network algorithm trained on lumbar spine radiographs to predict outcomes of transforaminal epidural steroid injection for lumbosacral radicular pain from spinal stenosis

**DOI:** 10.1038/s41598-024-59288-w

**Published:** 2024-04-11

**Authors:** Jeoung Kun Kim, Min Cheol Chang

**Affiliations:** 1 Department of Business Administration, School of Business, Yeungnam University, Gyeongsan-si, Republic of Korea; 2 Department of Physical Medicine and Rehabilitation, College of Medicine, Yeungnam University, 317-1, Daemyungdong, Namku, Daegu, 705-717 Republic of Korea

**Keywords:** Deep learning, Convolutional neural network, Lumbar spine, Spinal stenosis, Transforaminal epidural steroid injection, Computational biology and bioinformatics, Medical research

## Abstract

Little is known about the therapeutic outcomes of transforaminal epidural steroid injection (TFESI) in patients with lumbosacral radicular pain due to lumbar spinal stenosis (LSS). Using lumbar spine radiographs as input data, we trained a convolutional neural network (CNN) to predict therapeutic outcomes after lumbar TFESI in patients with lumbosacral radicular pain caused by LSS. We retrospectively recruited 193 patients for this study. The lumbar spine radiographs included anteroposterior, lateral, and bilateral (left and right) oblique views. We cut each lumbar spine radiograph image into a square shape that included the vertebra corresponding to the level at which the TFESI was performed and the vertebrae juxta below and above that level. Output data were divided into “favorable outcome” (≥ 50% reduction in the numeric rating scale [NRS] score at 2 months post-TFESI) and “poor outcome” (< 50% reduction in the NRS score at 2 months post-TFESI). Using these input and output data, we developed a CNN model for predicting TFESI outcomes. The area under the curve of our model was 0.920. Its accuracy was 87.2%. Our CNN model has an excellent capacity for predicting therapeutic outcomes after lumbar TFESI in patients with lumbosacral radicular pain induced by LSS.

## Introduction

Lumbar spinal stenosis (LSS) is caused by narrowing of the lumbar spinal canal or lumbar vertebral foramen^1^. LSS typically results from degenerative changes in the spine, including degeneration of the disc, formation of osteophytes, and thickening of spinal ligaments^1^. These degenerative changes cause the narrowing of space that is available for the neural and vascular elements in the lumbar spine^2^. LSS can cause pressure on the nerve roots and vascular structures^2^. In addition to the compression of neurovascular structures in the lumbar spine, LSS causes an inflammatory response in which various inflammation-mediated cells and proinflammatory cytokines are involved, resulting in lumbosacral radicular pain^3^. The radicular pain from LSS may be aggravated, particularly during walking or standing for long periods^4^.

Conservative treatments such as oral medication, physical therapy, and injection procedures are used to control the lumbosacral radicular pain caused by LSS^5,6^. Moreover, transforaminal epidural steroid injection (TFESI) is one of the most effective treatments for alleviating pain from LSS^3,6^. Corticosteroids inhibit the synthesis of various proinflammatory mediators^7^.

The prediction of therapeutic outcomes after TFESI is important because it allows clinicians to elucidate a therapeutic plan for lumbosacral radicular pain due to LSS. Previous studies have evaluated outcomes according to stenosis severity observed on magnetic resonance imaging (MRI)^3,8^. However, the prognostic evaluation methods of the previous studies only showed a tendency of the therapeutic outcomes and did not provide individualized outcomes based on the specific structural characteristics of each patient^3,8^. Furthermore, MRI is expensive and not easily accessible. We believe it is possible to assume the degree of spinal stenosis by assessing degenerative findings in lumbar spine radiographs, such as disc space narrowing, osteophyte formation, and facet degeneration^9^. Lumbar spine radiographs can be easily performed because almost all clinics and hospitals are equipped with a radiographic imaging machine, and the cost for patients is relatively low. However, at present, no study has analyzed the therapeutic outcomes of TFESI based on findings visible in lumbar spine radiographs.

Machine learning (ML) is a computer algorithm that can automatically learn from data without the need for explicit programming^10–12^. ML is known for its ability to overcome the limitations of existing image analysis techniques and enable breakthroughs in the field of image analysis^10–12^. Deep learning (DL) is an advanced ML approach that uses many hidden layers to build artificial neural networks with structures and functions similar to those of the human brain. It can learn from unstructured and perceptual image data, and several studies have demonstrated that the DL technique can outperform traditional ML techniques^13–15^. A convolutional neural network (CNN) is a representative DL model specializing in image analysis^16,17^. We believe that the CNN model can recognize and analyze the findings related to spinal degeneration on lumbar spine radiographs and could help predict the therapeutic outcome of TFESI. Furthermore, CNN can enable personalized prediction of therapeutic outcomes based on each patient’s images.

In the current study, we used lumbar spine radiographs as input data and trained a CNN model to predict therapeutic outcomes after lumbar TFESI in patients with lumbosacral radicular pain caused by LSS.

## Results

**Figure 1:**
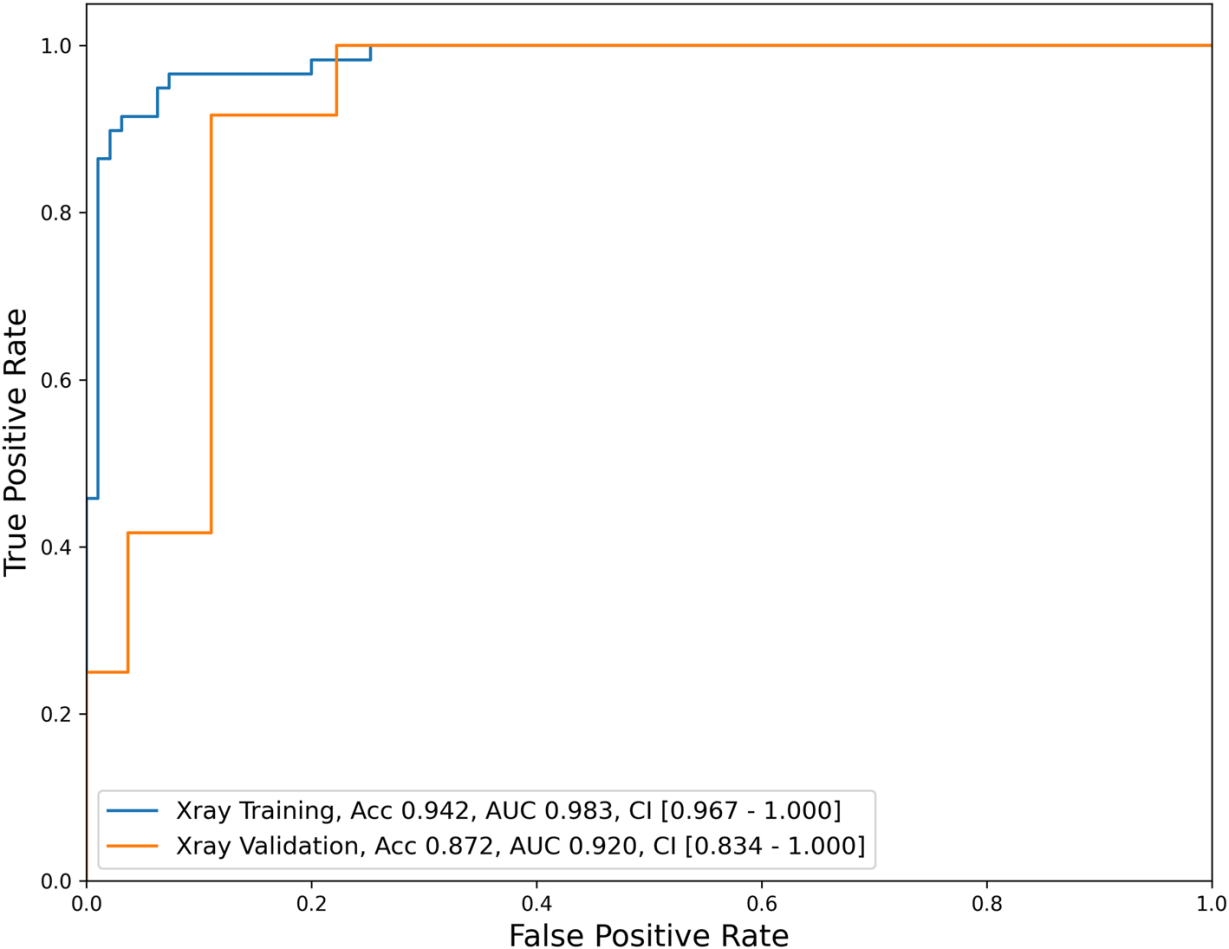
Receiver operating characteristic curves for the validation and test datasets of our developed model. Acc, accuracy; AUC, area under the curve; CI, confidence interval.

Table 1 summarizes the sample characteristics of the proposed model and its performance measures. This study utilized 193 samples, with a training set comprising 79.8% (154 samples) and a validation set comprising 20.2% (39 samples). The training set had a ‘favorable outcome’ to ‘poor outcome’ ratio of 38.3–61.7%, while the validation set had a ratio of 33.3–66.7%. The trained model demonstrated robust performance with a training accuracy of 94.2% and an AUC of 0.983 (95% CI [0.967–1.000]). The validation accuracy was also high at 87.2%, with an AUC of 0.920 (95% CI [0.834–1.000]) (Fig. 1).

**Table 1:** Model performance analysis.

	Developed model				
Sample size Sample ratio	154, 79.8% for training, 39, 20.2% for validation, total 193 Favor outcome: 59, 38.3%; Poor outcome: 95, 61.7% for training, total 154 Favor outcome: 12, 33.3%; Poor outcome: 27, 66.7% for validation, total 39				
Model details	- EfficientNetV2S CNN model with full training				
	- SGD optimizer, ReLU activation				
	- Learning rate 3e-05, batch size 16				
	- Batch normalization and dropout for regularization				
	- ROI image resized to (384 × 384)				
	- Specificity: 96.8% for training and 85.2% for validation				
	- Training accuracy: 94.2%, AUC 0.983 with 95% CI [0.967–1.000]				
	- Validation accuracy: 87.2%, AUC 0.920 with 95% CI [0.834–1.000]				

Model performance (validation data)	Class	Precision	Recall (sensitivity)	F1-score	Support
	Favor outcome (1)	0.733	0.917	0.815	12
	Poor outcome (0)	0.958	0.852	0.902	27
	Macro average	0.846	0.884	0.858	39
	Weighted average	0.889	0.872	0.875	39

CNN, convolutional neural network; SGD, stochastic gradient descent; ReLU, rectified linear units; ROI, region of interest; AUC, area under the curve; CI, confidence interval.

In terms of class-specific performance, for the ‘favorable outcome’ set, the precision was 0.733, recall was 0.917, and F1-score was 0.815. For the ‘poor outcome set’ the model showed a precision of 0.958, recall of 0.852, and F1-score of 0.902. The macro average across classes was a precision of 0.846, recall of 0.884, and F1-score of 0.858, while the weighted average was a precision of 0.889, recall of 0.872, and F1-score of 0.875.

These results suggest that the model is highly accurate and distinguishes between the ‘favorable outcome’ and ‘poor outcome’ with a particularly strong performance in identifying the ‘favorable outcome’. However, there is scope for improvement in the precision for the ‘favorable outcome’.

**Figure 2:**
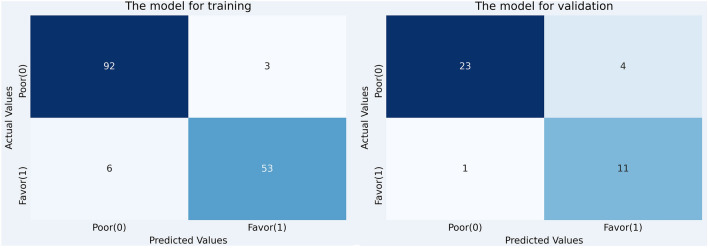
The confusion matrix for our developed model.

Figure 2 provides additional information on the model's characteristics through confusion matrix analysis for the validation data. The confusion matrix shows that the model correctly predicted 11 out of 12 patients who showed ‘favorable outcomes’ (91.7% precision). Also, the model correctly predicted 23 out of 27 patients with ‘poor outcomes’ (85.2% recall).

## Discussion

In our study, we developed a CNN algorithm for predicting therapeutic outcomes of lumbar TFESI in patients with lumbosacral radicular pain following LSS. The accuracy of our algorithm was 87.2%, and the AUC was 0.920. Considering that AUCs of 0.7–0.8, 0.8–0.9, and > 0.9 are considered as having acceptable, excellent, and outstanding diagnostic capacities, respectively, the ability of our CNN model that was developed using lumbar radiographs as input data seems to be excellent^18^.

While neural networks and other various algorithms have been utilized for the past 50 years, developments in the field of CNNs constitute significant accomplishments^14,17^. The multiple convolutional and pooling layers of the CNN algorithm enable the identification of radiologic features or other image-based data and assign weights to important features^16,17^. Also, the CNN algorithm is less affected by distortions, horizontal or vertical shifts, contrasts, angles, and partial masks in images; it also requires less computer memory, which allows for more effective algorithm training^19^. In accordance with these advantages of the CNN algorithm, our algorithm is expected to accurately identify key features influencing the therapeutic prognosis after TFESI in lumbar radiographs of patients with LSS.

Previous studies have evaluated therapeutic outcomes after TFESI in patients with LSS for alleviating lumbosacral radicular pain^3,8^. In 2018, Chang et al. evaluated the outcome of TFESI according to the severity of lumbar foraminal spinal stenosis (LFSS)^3^. Of 31 patients with mild to moderate LFSS, 27 patients (87.1%) showed favorable outcomes (≥ 50% pain reduction of initial pain at 3 months post-treatment). Of 26 patients with severe LFSS, 11 (42.5%) reported successful pain relief. In 2020, Do et al. evaluated the therapeutic outcomes of interlaminar epidural steroid injection in patients with chronic radicular pain according to the degree of lumbar central spinal stenosis (LCSS)^8^. At 3 months after TFESI, nine (30.0%) of 30 patients with moderate LCSS showed favorable outcomes (≥ 50% pain reduction of initial pain at 3 months post-treatment). Five (17.9%) of 28 patients with severe LCSS reported successful pain relief. However, these research results described only overall trends in therapeutic outcomes based on radiologic findings, such that it remains difficult to discern the favorable and poor therapeutic outcomes of an individual patient. In contrast, our algorithm can determine an individual’s therapeutic outcomes when inputting each patient’s lumbar radiographs into the algorithm.

Regarding studies that developed a DL algorithm to predict therapeutic outcomes after TFESI, to the best of our knowledge, two studies have been published^20,21^. In 2022, Kim et al. collected whole T2-weighted sagittal lumbar spine MR images from 503 patients with chronic lumbosacral radicular pain^20^. Similar to our study, the favorable and poor outcomes were defined as ≥ 50% and < 50% reduction at 2-month follow-up after TFESI, respectively. Kim et al. reported that the accuracy for predicting the therapeutic outcome of TFESI was 76.2%, and the AUC was 0.827. In 2023, Wang et al. recruited 288 patients with radicular pain due to cervical foraminal stenosis^21^. The authors collected single T2-axial spine MR images for each patient. They also defined ≥ 50% and < 50% reduction at 2-month follow-up after TFESI as favorable and poor outcomes, respectively. The accuracy of the developed model for predicting the outcome after TFESI was 79.3%, and the AUC was 0.801. Therefore, our study is the first to demonstrate the usefulness of a DL model trained using radiographs in predicting the therapeutic outcomes of spinal injections for radicular pain.

Integrating our research results with a cloud system could significantly enhance its accessibility and scalability. A cloud-based platform would allow for deployment of the developed model across healthcare settings, enabling real-time analysis of lumbar spine radiographs. This approach would facilitate a centralized database for training and updating the model with new data, improving its accuracy over time. Moreover, cloud integration supports collaborative efforts among healthcare professionals and researchers, allowing for seamless sharing of insights and advancements in the treatment of lumbosacral radicular pain caused by LSS. By leveraging cloud technology, the research outcomes can be made more widely available, offering optimized treatment plans on a global scale.

In conclusion, we found that a CNN model trained using four radiographs (the anteroposterior, lateral, and left and right obliques) per each patient had an excellent capacity (accuracy = 87.2%, AUC = 0.920) for predicting the therapeutic outcomes after lumbar TFESI in patients with lumbosacral radicular pain due to LSS. We believe that our developed model could be effectively applied as a supplementary tool in clinical practice by pain physicians. Our study had several limitations. (1) A relatively small number of patients were included. (2) We collected images from a single hospital. To increase the generalizability of our results, lumbar radiographs collected from multiple hospitals should be used as input data for the CNN training algorithm. (3) We assumed that the patients' pain was caused solely by single-level LSS. However, in reality, it is possible that the pain was associated with multiple levels of LSS. Therefore, for more accurate analysis, it is preferable to use the entire lumber spine image as input data for developing the DL algorithm. (4) We used only the NRS as output data. If functional data were used instead, the developed algorithm could provide more information. (5) For developing the DL algorithm, we used only lumbar spine radiographs as input data. Incorporating MRI data along with lumbar spine radiographs as input data could further improve the prediction accuracy of therapeutic outcomes after lumbar TFESI.

## Materials and methods

### Participants

This retrospective observational study involved 193 patients (mean age = 74.3 ± 9.8 years, men: women = 71:122, injection levels L3:L4:L5:S1 = 2:3:24:156:8, right: left: bilateral = 69:68:56) who visited the spine center of a university hospital and underwent lumbar TFESI for LSS between January 2013 and December 2021. The inclusion criteria for this study were as follows: (1) single-level lumbar TFESI for segmental pain that radiated to the lower extremity due to LSS; (2) ≥ 3 months history of a symptomatic lumbosacral radicular pain with > 3 on a numerical rating scale (NRS-11; 0 = no pain; 10 = the worst pain) prior to TFESI; (3) ≥ 50% temporary pain relief following a diagnostic nerve block with 1 mL of 2% lidocaine; and (4) MRI and electrophysiological findings corresponding to the clinical manifestations. The data of patients with a history of spinal surgery, such as lumbar fusion or laminectomy, before TFESI were excluded. The study protocol was approved by the institutional review board of Yeungnam university hospital, which waived the requirement for written informed consent owing to the retrospective nature of this study. The study was conducted in accordance with the Declaration of Helsinki.

### TFESI procedures

TFESI was conducted using the standard method described in a previous study. All injections were performed by a single interventional physiatrist specializing in spinal injections. A strict aseptic technique was used to perform the TFESI procedures. Patients were prone, and C-arm fluoroscopy (Siemens, Erlangen, Germany) was used to aid level identification and needle placement. Lidocaine 1% was administered at the needle insertion site, and the tip of a 25-gauge 90-mm spinal needle with a bend at the tip to allow for guidance was positioned between the lateral vertebral body and the 6 o’clock position below the pedicle. Lateral fluoroscopic imaging demonstrated the presence of the needle tip between the spinal laminar margin and the posterior vertebral body. Under anteroposterior fluoroscopy, 0.3 mL of non-ionic contrast material was injected to confirm the absence of vascular uptake and spread of contrast into the foramen. Subsequently, another injection of the contrast medium was performed under real-time fluoroscopic monitoring, and 20 mg (0.5 mL) of triamcinolone with 0.5 mL of bupivacaine hydrochloride and 1 mL of normal saline was injected.

### Images used for the DL algorithm (input data)

Lumbar spine radiographs were used as input data for developing the DL algorithm. Lumbar spine radiographs that were used as input data include the anteroposterior, lateral, and bilateral oblique (left and right oblique) views. Oblique lumbar radiographs were obtained from a 45° anteroposterior orientation on both the left and right sides of each patient. Additionally, we cut each lumbar spine radiograph image into a square shape that included the vertebra corresponding to the level at which the TFESI was performed, as well as the vertebrae juxta below and above that level.

To prepare data for DL, the region of interest (ROI) was isolated, with images segmented by a physiatrist to delineate regions with critical lesions, and image dimensions were standardized. This ROI protocol positively influenced the learning efficacy of the DL model. Furthermore, image features were normalized prior to their input into the CNN model to optimize generalization capability. This approach enhances applicability of the model across diverse datasets within the medical imaging domain. Medical imaging requires precise lesion detection, therefore attributes such as brightness adjustment, blurring, and noise were not utilized in image processing methodology in this study.

### Measurement of therapeutic outcome (output data)

Pain severity at pretreatment and 2-month follow-up after TFESI was assessed on the NRS (0 = no pain; 10 = worst pain). The NRS data were collected via chart review. A “favorable outcome” was defined as a ≥ 50% reduction in NRS score at 2 months post-TFESI versus the pretreatment NRS score. A “poor outcome” was defined as a < 50% reduction in NRS score at 2 months post-TFESI versus the pretreatment score. To validate the change in pain reduction, NRS scores were evaluated by assessing the difference between the pretreatment NRS scores and the 2-month post-treatment scores (change in NRS [%] = [pretreatment score − 2 months post-TFESI score]/pretreatment score × 100).

### DL algorithms

**Figure 3:**
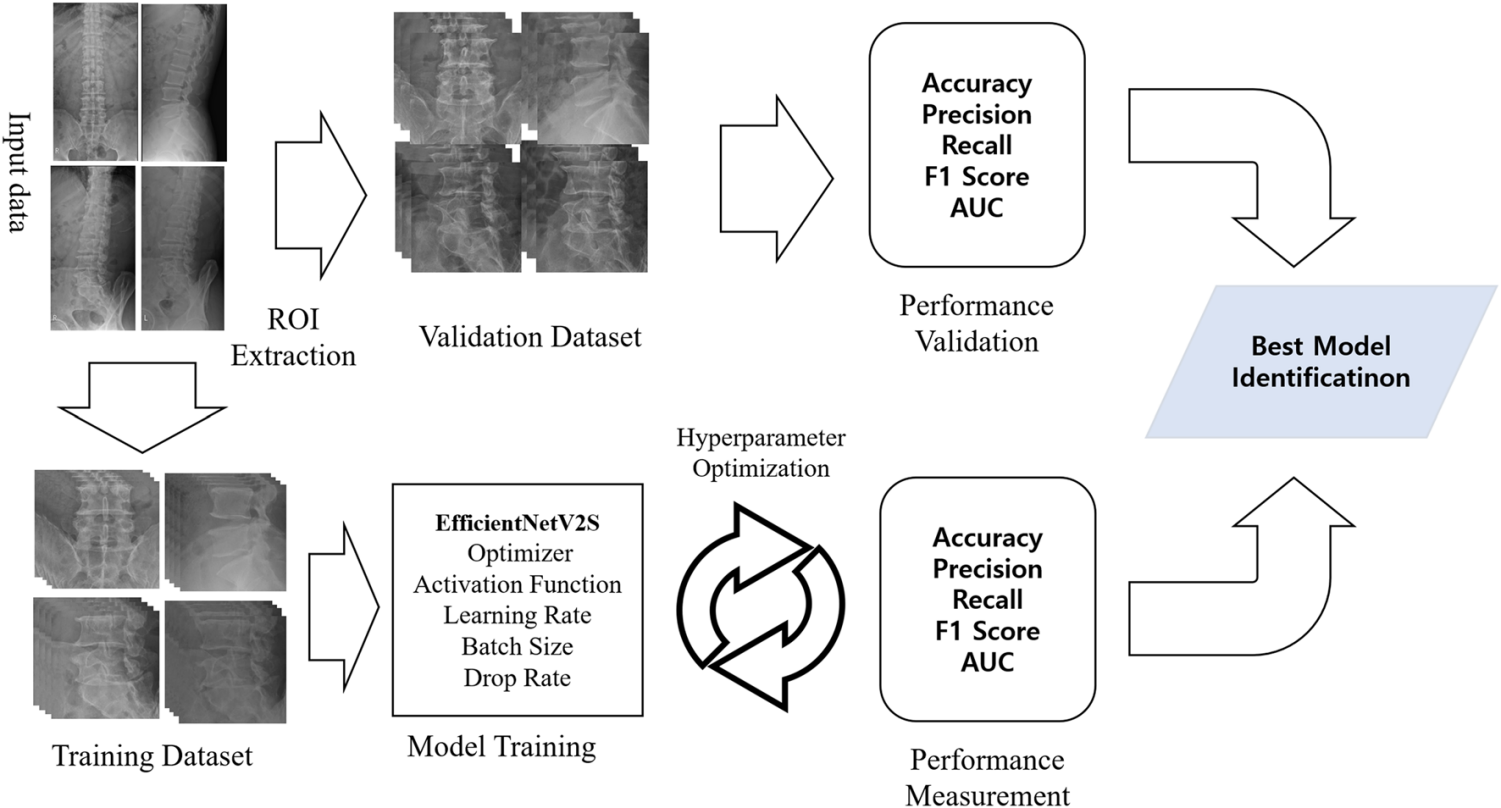
Diagram of the process for the development of the deep learning model for predicting the therapeutic outcome after transforaminal epidural steroid injection in patients with lumbosacral radicular pain due to lumbar spinal stenosis. ROI, region of interest; AUC, area under the curve.

Python 3.8.10, scikit-learn 1.1.2, and TensorFlow 2.13.0 with Keras were used to develop the CNN model for predicting TFESI outcomes. We concurrently fed four X-ray ROI images (anteroposterior, lateral, left oblique, and right oblique) into the EfficientNetV2S CNN model for training. We employed a range of optimizers, learning rates, and batch sizes, integrating dropout regularization techniques to mitigate overfitting. Table 2 provides detailed information about the proposed model. The table is based on the outputs generated using TensorFlow’s model.summary() function. Figure 3 offers a concise summary of each phase within the model training procedure.

**Table 2:** TFESI model detail.

Layer (type)	Output Shape	Param #
anteroposterior img (InputLayer)	[(None, 384, 384, 3)]	0
lateral img (InputLayer)	[(None, 384, 384, 3)]	0
left oblique img (InputLayer)	[(None, 384, 384, 3)]	0
right oblique img (InputLayer)	[(None, 384, 384, 3)]	0
efficientnetv2-s (Functional)	(None, None, None, 1280)	2,033,136
global_average_pooling2d (GlobalAveragePooling2D)	(None, 1280)	0
global_average_pooling2d_1 (GlobalAveragePooling2D)	(None, 1280)	0
global_average_pooling2d_2 (GlobalAveragePooling2D)	(None, 1280)	0
global_average_pooling2d_3 (GlobalAveragePooling2D)	(None, 1280)	0
concatenate (Concatenate)	(None, 5120)	0
dense (Dense)	(None, 1024)	5,243,904
batch_normalization (BatchNormalization)	(None, 1024)	4,096
Dropout (Dropout)	(None, 1024)	0
Dense_1 (Dense)	(None, 1024)	1,049,600
batch_normalization_1 (BatchNormalization)	(None, 1024)	4,096
dense_2 (Dense)	(None, 1)	1,025
Total params: 26,634,081 (101.60 MB) Trainable params: 26,476,113 (101.00 MB) Non-trainable params: 157,968 (617.06 KB)

### Statistical analysis

Statistical analyses were executed utilizing Python 3.8.10 and scikit-learn version 1.1.2. A receiver operating characteristic (ROC) curve analysis was conducted, and the area under the curve (AUC) was computed. The 95% confidence interval (CI) for the AUC was determined following the method outlined by DeLong et al.^22^. Scikit-learn was employed for computing both the ROC curve and AUC. The classification report function in scikit-learn was employed to compute the accuracy, class-specific precision, recall, and F1 score.

## Data Availability

Some or all data, models, or code generated or used during the study are available from the corresponding author by request.
